# Genome Sequence of *Luteimonas huabeiensis* HB-2, a Novel Species of *Luteimonas* with High Oil Displacement Efficiency

**DOI:** 10.1128/genomeA.00152-14

**Published:** 2014-03-06

**Authors:** Yang Liu, Su Yao, Yong Liu, Youqiang Xu, Chi Cheng

**Affiliations:** a China National Research Institute of Food and Fermentation Industries, Beijing, People’s Republic of China; b China Center of Industrial Culture Collection, Beijing, People’s Republic of China

## Abstract

*Luteimonas huabeiensis* HB-2 is a novel and newly isolated strain, which shows a superior property of oil displacement. Here, we present a 4.3-Mb assembly of its genome. The key genes for phospholipid and fatty acid metabolism were annotated, which are crucial for crude oil emulsification and recovery.

## GENOME ANNOUNCEMENT

Adepleting crude oil supply is becoming the overriding challenge faced by oil industries (1). In the process of oil exploration, much residual oil became trapped in rock pores after the first and second oil explorations in China (2). Microbial enhanced oil recovery (MEOR) has been a useful technology, which enhances the recovery of crude oil from reservoir rocks due to the metabolic products synthesized by microorganisms (3). *Luteimonas huabeiensis* HB-2 is a novel species of *Luteimonas* newly isolated from stratum water located in the Huabei Oil Field, China (4). This strain shows a superior ability for enhanced oil recovery, which might increase the recovery ratio of crude oil about 15.4%, as seen in laboratory experiments (our unpublished data).

Here, we announced the draft genome sequence of strain HB-2. The genome sequence was determined by the Illumina HiSeq 2000 (5). The genome sequence contains 5,474,430 reads for shotgun sequencing and 11,810,420 reads for paired-end sequencing. The reads were assembled into 86 contigs (>400 bp), with a length of 4,295,921 bp and a G+C content of 71.6%, using the CLC Genomics Workbench 5.0.1 system (CLC bio, Aarhus, Denmark). The genome sequence was annotated by the RAST server (6). tRNAs and rRNAs were predicted by tRNAscan-SE version 1.23 (7) and RNAmmer 1.2 (8), respectively.

The genome sequence of strain *L. huabeiensis* HB-2 contains 3,894 protein-coding sequences (CDSs). Forty-six RNAs were identified, including 45 tRNAs and one rRNA. Twenty-nine CDSs for phospholipid metabolism and 48 CDSs for fatty acid metabolism were annotated. Phospholipid and fatty acids have been reported to be surfactants (9, 10), which play important roles in oil emulsification and reducing oil viscosity. The genome sequence also contains three CDSs for dioxygenase. Previous studies showed that dioxygenase is important for the ring cleavage of aromatic compounds in oil (11, 12), which is responsible for the degradation of aromatic compounds in oil. The genome sequence and annotation of *L. huabeiensis* HB-2 might promote further research about the molecular mechanisms of oil recovery and contribute to the enhancement of its oil displacement capability.

### Nucleotide sequence accession number.

This whole-genome shotgun project has been deposited at GenBank under the accession no. JAAN00000000. The version described in this paper is the first version.
